# Assistant Diagnosis of Basal Cell Carcinoma and Seborrheic Keratosis in Chinese Population Using Convolutional Neural Network

**DOI:** 10.1155/2020/1713904

**Published:** 2020-08-01

**Authors:** Kai Huang, Xiaoyu He, Zhentao Jin, Lisha Wu, Xinyu Zhao, Zhe Wu, Xian Wu, Yang Xie, Miaojian Wan, Fangfang Li, Dihui Liu, Nianzhou Yu, Mingjia Li, Juan Su, Shuang Zhao, Xiang Chen

**Affiliations:** ^1^Hunan Engineering Research Center of Skin Health and Disease, Changsha, Hunan, China; ^2^Hunan Key Laboratory of Skin Cancer and Psoriasis, Changsha, Hunan, China; ^3^Dermatology Department of Xiangya Hospital of Central South University, Changsha, Hunan, China; ^4^School of Automation, Central South University, Changsha, Hunan, China; ^5^Tencent Medical AI Lab, Beijing, China; ^6^Department of Dermatology, The 3^rd^ Affiliated Hospital of Sun Yat-Sen University, Guangzhou, China; ^7^Xiangya School of Medicine, Central South University, Changsha, Hunan, China

## Abstract

**Objectives:**

To evaluate CNN models' performance of identifying the clinical images of basal cell carcinoma (BCC) and seborrheic keratosis (SK) and to compare their performance with that of dermatologists.

**Methods:**

We constructed a Chinese skin diseases dataset which includes 1456 BCC and 1843 SK clinical images and the corresponding medical history. We evaluated the performance using four mainstream CNN structures and transfer learning techniques. We explored the interpretability of the CNN model and compared its performance with that of 21 dermatologists.

**Results:**

The fine-tuned InceptionResNetV2 achieved the best performance, with an accuracy and area under the curve of 0.855 and 0.919, respectively. Further experimental results suggested that the CNN model was not only interpretable but also had a performance comparable to that of dermatologists.

**Conclusions:**

This study is the first on the assistant diagnosis of BCC and SK based on the proposed dataset. The promising results suggested that CNN model's performance was comparable to that of expert dermatologists.

## 1. Introduction

Basal cell carcinoma (BCC) and seborrheic keratosis (SK) are benign and malignant skin tumors, respectively [1]. Generally, patients with BCC require surgery for complete tumor resection, while most patients with SK do not need special treatment and only need to maintain observation [2, 3]. Therefore, the identification of the two skin diseases is clinically significant.

Clinically, the identification of BCC and SK needs to take the appearance of skin lesions, medical history, and results of biopsy into consideration. The biopsy can effectively identify these two types of diseases, whereas it is an invasive examination and requires professional pathologists to operate (many hospitals often lack pathologists to perform biopsy). As a result, a noninvasive diagnostic method needs to be proposed for the identification of BCC and SK.

With the rapid development of artificial intelligence, convolutional neural networks (CNNs) have been extensively applied for feature learning [4, 5], image classification [6, 7], and object detection [8, 9]. In the field of medical image analysis tasks, CNNs have been widely utilized to classify skin diseases and even reach the level of professional dermatologists in some tasks, which is considered by most researches [10–14]. Esteva et al. [10] trained on about 130,000 clinical images by using the CNN architecture of Inceptionv3 and verified that the performance of the CNN models could be comparable to that of professional dermatologists in the task of classifying malignant melanoma and benign nevus. Han [11] trained a classification model on 19,398 clinical images (covering 12 categories of skin tumors) using the CNN structure of Resnet152. The average AUC of this model can reach 0.91, which is verified to be comparable to 16 professional dermatologists. Tschandl et al. [12] proposed a novel network (named cCNN) to realize the diagnosis of nonpigmented skin cancers, which can take both images of dermoscopy and clinical images as input. They trained a classification model on 7,895 images of dermoscopy and 5,829 clinical images and tested it on a set of 2,072 unknown cases. The trained CNN model was suggested to classify nonpigmented lesions as accurately as human experts. Haenssle et al. [13] used the pretrained InceptionV4 to train the classification model for identifying melanoma and benign melanocytic nevus on 100,000 dermatoscopic images. The diagnostic performance of their CNN model was compared with 58 dermatologists from all over the world. The results showed that the diagnostic level of CNN models reached or even exceeded that of professional dermatologists. Maron et al. [14] used the ResNet50 CNN architecture to train a multiclassification model on 12,336 images of dermoscopy. The diagnostic performances of the CNN model and 112 dermatologists were evaluated on 300 biopsy-verified images of dermoscopy from two standpoints of benign and malignant classification and multilevel task classification. At the equal sensitivity of 74.4% and 56.5%, the specificity of the CNN model is 91.3% and 98.8%, which are both higher than the dermatologists, 59.8% and 89.2%. However, most of the previously mentioned research studies are about the classification of images of dermoscopy of several common skin tumors, especially the task of melanoma or nonmelanoma. Until now, we have not found any studies related to the clinical image classification of BCC and SK in Chinese population.

In this paper, we conducted the first study for the assistant diagnosis of BCC and SK in Chinese population. The main contributions of this paper were summarized as follows:

1. A Chinese skin disease dataset of BCC and SK was constructed with the help of dermatologists in Xiangya Hospital, Central South University. the label of each image was confirmed by the pathological examination and the whole dataset together with the labels will be publicly available for academic research.
2. Inspired by transfer learning strategies, the performance of four mainstream CNN architectures, including InceptionV3 [15], InceptionResNetV2 [16], DenseNet121 [17], ResNet50 [18], were compared on the task of classifying clinical images of BCC and SK.
3. To explore the interpretability of the diagnostic model, three methods were implemented, including t-distributed Stochastic Neighbor Embedding (t-SNE) [19], Gradient-weighted Class Activation Mapping (Grad-CAM) [20], and Local Interpretable Model-Agnostic Explanations (LIME) [21].
4. To evaluate the real-world performance of the diagnostic model, we compared the performance between the fine-tuned InceptionResNetV2 and 21 dermatologists on the additional test set of 116 subsequently collected clinical images.

## 2. Methods

### 2.1. Data Collection and Processing

**Figure 1:**
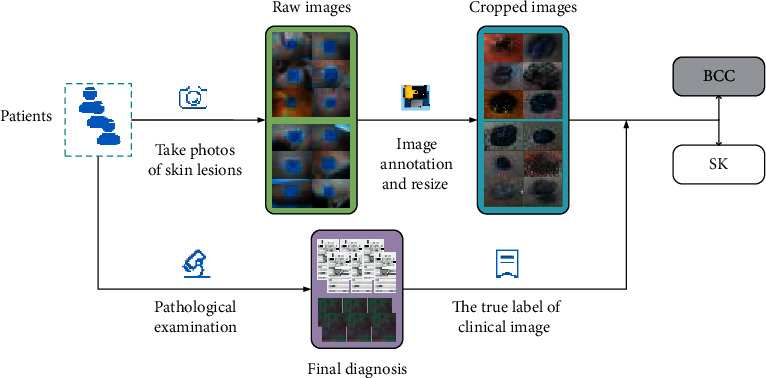
Workflow of the dataset collection and annotation.

**Figure 2:**
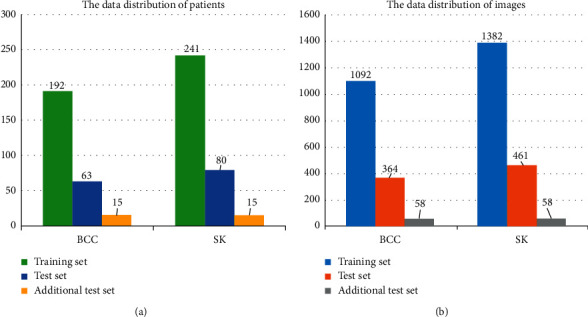
The data distribution of the training set, test set, and additional test set.

With the informed consent of each patient and the approval of the ethical committee of Xiangya Hospital, Central South University, we have been collecting the clinical image dataset from January 2016 to December 2018. To acquire a detailed visual representation of BCC and SK, we used a high-definition digital camera (SONY DSC-HX50, 350 dpi) to obtain high-quality clinical images with a resolution of 5120 *∗* 3480, in which the number of BCC and SK was 541 and 684, respectively, and whose diagnostic label was verified by the pathological examination. In order to remove the complex background of the clinical images, 12 dermatologists were asked to annotate the specific location of the skin lesions using an open-source software called LabelImg (https://tzutalin.github.io/labelImg/). In this process, some bad images that may affect CNN's learning process were filtered out, including blurred images, skin lesions covered with hair or other visual elements, skin lesions altered by visible topical treatments, and so on. Then, we used these annotation files to crop the image and adjust image resolution to 300 *∗* 300. Finally, our dataset consisted of 1456 BCC and 1843 SK images, each of which was equipped with the corresponding medical history and pathological diagnosis. The workflow of the dataset collection and annotation is shown in Figure 1. Then, we randomly divided the cropped images into the training set and test set according to different patients with the ratio of 3 : 1, ensuring that the same patient's images will not appear in both training set and test set. In detail, we divided the training set into training portion and verification portion, with the ratio of 3 : 1. The utilization of the verification portion is to evaluate the performance of the model during the training process. In addition, we collected an additional test set of 116 clinical images from January 2019 to June 2019, which will be used as the test set for the comparison between CNN model and dermatologists. The numbers of patients and images in the training set, test set, and additional test set are shown in Figure 2.

### 2.2. Binary Classifier of BCC and SK

In this paper, we used four mainstream CNN networks to build the binary classifier of BCC and SK, including InceptionV3 [15], InceptionResNetV2 [16], DenseNet121 [17], and ResNet50 [18]. The detailed introduction and the differences of four CNN structures can be seen in Appendix in Supplementary Materials 1. In order to apply these CNN structures to our BCC and SK classification task, we set the output dimension of the last fully connected layer to 2. On the training set, we compared the CNN training means of fine-tuning from the pretrained weights and training from scratch; that is, eight models were compared in this experiment in total. We kept the same experimental parameters by setting the same image batch size of 30, max iteration epochs of 100, optimizer of root mean square prop (RMSProp), and loss function of cross entropy. All experiments were conducted on three graphic processing units of Nvidia Titan XP. After the training process, these models were tested on the same test set and evaluated by the performance indexes of ACC, ACCAvg, Sen, Spe, and AUC.

### 2.3. Model Interpretability

In this paper, we implemented three methods to enhance the interpretability of our BCC and SK diagnostic model including t-SNE [19], Grad-CAM [20], and LIME [21]. The t-SNE is a nonlinear dimensionality reduction algorithm in machine learning, which is very suitable for reducing high-dimensional data to 2D or 3D for visualization. We mainly used t-SNE to visualize the features of the upper layer of Softmax in the CNN structure. Grad-CAM can use the gradient information of the last convolutional layer in the CNN to highlight essential regions of the input image that are important for the prediction category. LIME can also be used to generate the corresponding saliency map for the input image, and it can be applied to any classification models in machine learning, not limited to CNNs. Therefore, we used Grad-CAM and LIME together to view the classification basis of the CNN diagnostic model.

### 2.4. CNN vs Dermatologists

To compare the diagnostic capacity between the CNN model and dermatologists, we have conducted a comparative experiment on the additional test set. The 21 participating dermatologists were divided into two different levels according to China's physician training and professional title evaluation system, including 8 expert dermatologists and 13 general dermatologists. General level refers to the trainees who have successfully finished the standardized training of resident doctors for three years and attending doctor, and expert level refers to the level of doctors who have obtained the title of associate chief physician and above. These dermatologists were asked to read each case of the additional test set one by one on a website (http://sugurs.gz01.bdysite.com/bccsk/) and give the answer (BCC or SK) in no more than 30 seconds. The backstage of the website will record the participants' answers so that we can calculate the diagnostic performance of each dermatologist. For the CNN, we simply used the CNN model to test on the total additional test set and calculated the diagnostic performance by comparing the CNN model's prediction results with the ground truth.

## 3. Results

### 3.1. Binary Classifiers of BCC and SK

We compared four CNN networks in training mean of fine-tuning with the pretrained weights on ImageNet and training from scratch. The detailed test results are summarized in Table 1. We labeled the highest value for each performance index in both two training means. In terms of AUC, the four models fine-tuned with the pretrained weights on ImageNet outperformed those that were trained from scratch (InceptionV3: 0.896 vs 0.894, InceptionResNetV2: 0.919 vs 0.895, DenseNet121: 0.913 vs 0.890, and ResNet50: 0.905 vs 0.879). However, this kind of improvement of four CNN models does not appear in the performance index of average classification accuracy. It is worth noting that AUC, an indicator that describes the confidence of the prediction results, is commonly considered to be a more important performance index for binary classifiers rather than classification accuracy [22]. By comparing four CNN models that all take fine-tuning techniques, the best performance is achieved by InceptionResNetV2 with the highest AUC of 0.919, and the average accuracy of 0.855 is also the best among four models as well.

### 3.2. Model Interpretability

**Figure 3:**
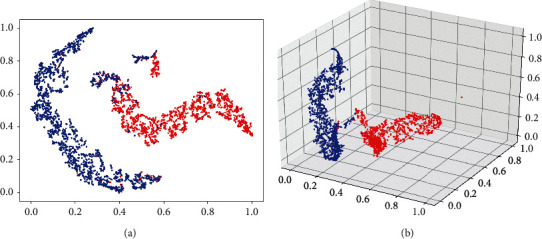
The visualization results of the features extracted by CNN. (a) Reduction of the 1536-dimensional features to two-dimensional. (b) Reduction of the 1536-dimensional features to three-dimensional.

**Figure 4:**
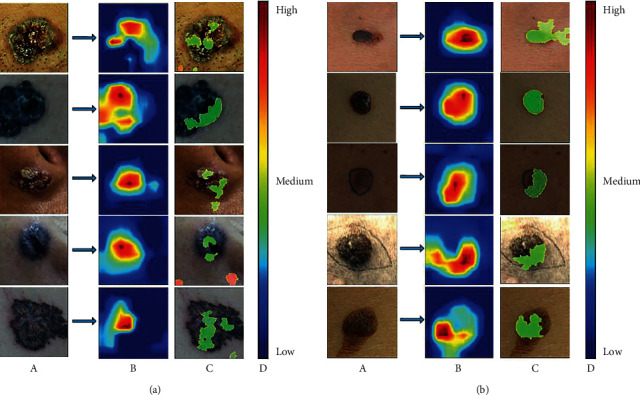
Results of the saliency maps. (a) The test results of BCC. (b) The test results of SK. (A) The input clinical images. (B) The saliency maps of the input images generated by Grad-CAM. (C) The saliency maps of the input images generated by lime. (D) The scale color bar indicates the importance of the corresponding position from low to high.

In this paper, we selected the fine-tuned InceptionResNetV2 which had the best performance in the last experiment to implement the model interpretability methods, including t-SNE, Grad-CAM, and LIME. The results of t-SNE are shown in Figure 3. Each point in this figure represents a sample in the dataset, and the red and blue points represent BCC and SK, respectively. The function of t-SNE is to reduce the output of the 1536-dimensional features from InceptionResNetV2 to two-dimensional and three-dimensional. The results of Grad-CAM and LIME are shown in Figure 4. For the results of Grad-CAM, the color of the pixel from dark blue to red indicates the importance from lowest to highest. For the results of LIME, the green and red super-pixel blocks denote a supported and unsupported area for the prediction result of the model, respectively.

### 3.3. CNN vs Dermatologists

**Figure 5:**
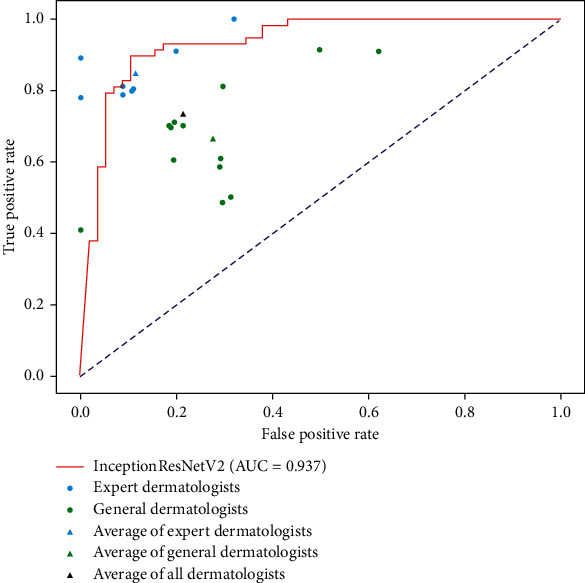
The ROC curve of the comparative experiment between the diagnostic performance of CNN and that of dermatologists.

The results of the comparison between CNN and dermatologists are shown in Figure 5. The red curve represents the ROC curve of the CNN model which consists of a series of value of true positive rate (TPR) and false positive rate (FPR) at different thresholds. The TPR and FPR of 8 expert dermatologists were 0.897, 0.793, 0.793, 0.793, 0.793, 0.897, 1.000 and 0.000, 0.000, 0.103, 0.103, 0.103, 0.207, 0.293, with an average of 0.845 (TPR) and 0.114 (FPR). Moreover, the TPR and FPR of 13 general dermatologists were 0.793, 0.707, 0.707, 0.707, 0.707, 0.603, 0.603, 0.603, 0.897, 0.500, 0.500, 0.397, 0.897 and 0.293, 0.207, 0.207, 0.207, 0.207, 0.207, 0.293, 0.293, 0.500, 0.293, 0.293, 0.000, 0.603, with an average of 0.663 (TPR) and 0.277 (FPR). The average TPR and FPR of 21 dermatologists were 0.732 and 0.215, of which the best TPR and FPR were 0.897 and 0, respectively. The blue and green dots represent the diagnostic results of the expert and general dermatologists, respectively. From Figure 5, we could see that the InceptionResNetV2 model could result in an AUC of 0.937, and the optimal TPR and FPR are 0.897 and 0.103, which outperforms the average of 13 general dermatologists and is comparable to the average of 8 expert dermatologists.

## 4. Discussion

Currently, in the field of assistant diagnosis of skin tumors, there is an urgent lack of datasets for Chinese populations. This paper takes the lead in putting forward the first Chinese race dataset of BCC and SK. In our dataset, each image has a corresponding pathological examination to ensure the authenticity of the label.

In the comparative experiment between the CNN model and 21 dermatologists, the results show that the constructed CNN model is superior to the average of the general dermatologists in the task of diagnosing BCC and SK and is comparable to the expert dermatologists. However, there is still a certain gap with the best doctors (TPR: 0.897, FPR: 0). Similarly, the same conclusions can be derived through other researches. Note that these results only show that CNN model is superior to dermatologists in the ability of clinical image classification. However, from the perspective of clinical practice, since this experiment only considers the image as an input, it seems unfair to the dermatologists who usually need to take patient's gender, age, lesion location, medical history, and results of pathological examination together to make the final decision. For example, the study by Haenssle et al. [13] has shown that multimodal information can improve the diagnostic ability of dermatologists. In addition, research by Tschandl et al. [12] has shown that multimodal information can enhance the diagnostic performance of CNN models. Consequently, we will focus our attention on the multimodal researches on skin tumors in the future, for the purpose of imitating the dermatologists' diagnosis process as much as possible.

Although CNN has made great achievements in the field of medical image analysis, the existing studies always take CNN as a black box, without further revealing the classification basis of the CNN model. The lack of model interpretability severely limits the applications of CNN in medical diagnosis. To address this issue, we conducted model interpretability studies. Figure 3 shows that CNN model could effectively separate BCC and SK samples due to CNN's capacity of deep feature extraction. Different from the work of Han et al. [11], who carried out visual interpretability research by using Grad-CAM, we used Grad-CAM together with lime to conduct CNN interpretability research. Figure 4 shows that both two methods are generally able to locate the lesion areas of the input images, while the visualization results are not the same due to the different principles of the methods. The implementation of Grad-CAM includes deconvolution and depooling, resulting in some position offsets. For LIME, it searches for important areas for the prediction result based on the super-pixel blocks of the input image, which can be used in any classification models regardless of the specific structure of the model. Comparing these two methods, we found that LIME results are more direct and accurate, which can even be used as the location selection of skin biopsy.

**Figure 6:**
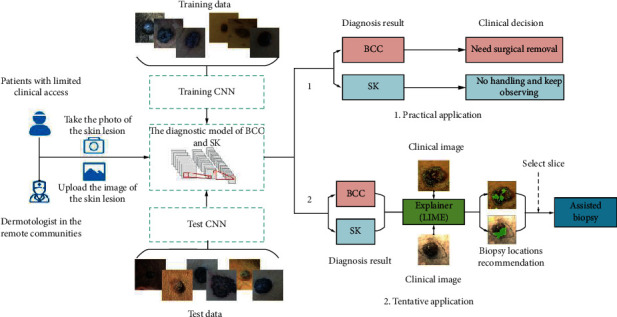
Applications of the proposed diagnostic model in the clinical practice.

In China, there are only 22,000 registered dermatologists with a population of about 1.3 billion, the ratio of which is about 1 : 60,000. More seriously, about 800 million individuals live in rural areas, but 80% of the medical institutions with high-quality medical resources are concentrated in cities [23]. Therefore, the establishment of the computer-aided diagnostic system in areas short of dermatologists is of great clinical significance. In conclusion, the diagnostic model explored in this paper will have the following two applications: practical application and tentative application (Figure 6). The first application is to assist dermatologists in the remote community to make clinical decisions. The dermatologist can take a clinical picture from the patient and get a predicting result through this model. For patients with a diagnosis of BCC, they need to go to a high-level hospital for surgical resection, while, for patients of SK, what they should do is only to keep watching it (waiting for observation and treatment). The second possible application is the recommendation of the biopsy locations. We all know that the proper choice of biopsy location can effectively reduce the incidence of missed diagnosis, but this operation requires the dermatologists with diagnostic experience. Inspired by the model interpretability experiments, LIME, which can produce important visual clues for input images (see Figure 4), can be applied to generate the recommendation of the biopsy locations.

## 5. Limitation

There are exactly some limitations in this paper. First, the quantity of the clinical images of BCC and SK is not large, so more pictures need to be collected to verify the generalization performance of the model. Second, our model only takes the clinical images as the input, without considering the information of the patient metadata, for example, gender, age, and lesion location. Multimodal data can reflect the real world more and can be used in practice. This is also our next step. In addition, for the introduced application of recommending biopsy location, due to the lack of clinical trials, whether this application is feasible or not is yet to be verified by actual cases.

## 6. Conclusion

In this paper, we have proposed the first study of the diagnosis of BCC and SK in Chinese population and compared the diagnostic performance between the CNN model and dermatologists. First, we constructed a skin disease dataset based on Chinese population, including 1456 and 1843 high-quality clinical images of BCC and SK. Second, we evaluated the classification performance of four mainstream CNN networks training from scratch and using the fine-tuning technique. On the test set, the fine-tuned InceptionResNetV2 resulted in the best classification performance. Third, we implemented three methods and demonstrated the effectiveness of the deep feature extraction and visual clues for the model's predicting result. Finally, on the additional test set, we suggested that the performance of the CNN model can be comparable to that of expert dermatologists. In the future, we will collect more samples of BCC and SK to verify the generalization performance of this model, consider increasing patient metadata as the model input, and finally verify the consideration for the recommendation of biopsy location through clinical trials.

## Figures and Tables

**Table 1:** Test results of four CNN models training from scratch and fine-tuning from the pretrained weights. Here, ACC, ACC_Avg_, Sen, Spe, and AUC denote accuracy, average accuracy, sensitivity, specificity, and area under the curve, respectively.

Training means	Model	Diagnosis	ACC	ACC_Avg_	Sen	Spe	AUC
Scratch	InceptionV3	BCC	0.758	0.840	0.852	0.846	0.894
		SK	0.905		0.846	0.852	
	InceptionResNetV2	BCC	0.854	0.856	0.854	0.859	0.895
		SK	0.857		0.859	0.854	
	DenseNet121	BCC	0.835	0.844	0.846	0.848	0.890
		SK	0.850		0.848	0.846	
	ResNet50	BCC	0.786	0.834	0.783	0.883	0.879
		SK	0.872		0.883	0.783	
Fine-tuning	InceptionV3	BCC	0.857	0.836	0.852	0.837	0.896
		SK	0.820		0.837	0.852	
	InceptionResNetV2	BCC	0.775	0.855	0.791	0.915	0.919
		SK	0.918		0.915	0.791	
	DenseNet121	BCC	0.871	0.846	0.885	0.816	0.913
		SK	0.826		0.816	0.885	
	ResNet50	BCC	0.758	0.845	0.808	0.894	0.905
		SK	0.913		0.894	0.808	

## Data Availability

The dataset of the study is shared at http://airl.csu.edu.cn/xiangyaderm/.

## Supplementary Materials

Supplementary Materials Appendix 1: this file describes the differences among the four mainstream CNN structures including InceptionV3, InceptionResNetV2, DenseNet121, and ResNet50.
