# Supplementary material for: Assistant Diagnosis of Basal Cell Carcinoma and Seborrheic Keratosis in Chinese Population Using Convolutional Neural Network
Source: J Healthc Eng. 2020 Aug 1;2020:1713904. doi: 10.1155/2020/1713904 (PMC7422221; doi:10.1155/2020/1713904)
Supplement: Supplementary Materials — Appendix 1: this file describes the differences among the four mainstream CNN structures including InceptionV3, InceptionResNetV2, DenseNet121, and ResNet50. [file 1713904.f1.docx]

**1. Detailed architectures of four** **CNN architectures**

**2. Introduction of four CNN architectures and their characteristics**

- The InceptionV3 network was proposed by Google. Firstly, it introduced the idea of factorization into small convolutions, which is represented by decomposing a large two-dimensional (7*7) convolution kernel into two one-dimensional convolution kernels (1*7 and 7*1). In addition, Inception V3 optimizes the structure of the Inception Module and uses branches in the Inception Module. Therefore, Inception V3 can save a lot of network parameters, speed up parameter updates, and reduce overfitting.
- The InceptionResNet network combines the advantages of the Inception network and the residual network, which can deepen the network level, reduce network parameters, speed up model training, and greatly improve network performance.
- Densenet network adopts dense connection inside each Dense Block, while the traditional convolution layer and pooling layer are used between adjacent Dense Block. Dense connection is a direct connection between any two layers in the Dense Block. The input of each layer in the network is the union of the outputs of all the previous layers. At the same time, the output characteristics of each layer will be directly used as the input of the subsequent layers. Through dense connections, the problem of gradient disappearance can be alleviated, feature propagation can be enhanced, feature reuse can be encouraged, and the amount of parameters can be greatly reduced.
- The residual network introduces the output features of the first few layers to the subsequent network layer as an input part by skipping multiple layers directly, so the input features of the subsequent network layer will be linearly contributed by the input of a previous layer. Experiments have proved that the deep residual network can overcome the problem that the learning efficiency becomes lower and the accuracy cannot be effectively improved due to the deepening of the network, thereby deepening the network level.
